# Widespread Elevational Occurrence of Antifungal Bacteria in Andean Amphibians Decimated by Disease: A Complex Role for Skin Symbionts in Defense Against Chytridiomycosis

**DOI:** 10.3389/fmicb.2018.00465

**Published:** 2018-03-14

**Authors:** Alessandro Catenazzi, Sandra V. Flechas, David Burkart, Nathan D. Hooven, Joseph Townsend, Vance T. Vredenburg

**Affiliations:** ^1^Department of Biological Sciences, Florida International University, Miami, FL, United States; ^2^Department of Zoology, Southern Illinois University, Carbondale, IL, United States; ^3^Department of Biological Sciences, Universidad de Los Andes, Bogotá, Colombia; ^4^Department of Biology, San Francisco State University, San Francisco, CA, United States

**Keywords:** 16S rRNA gene, amphibian declines, amphibian skin bacteria, antifungal bacteria, elevational gradient, montane diversity gradient, neotropical, tropical Andes

## Abstract

Emerging infectious disease is a growing threat to global health, and recent discoveries reveal that the microbiota dwelling on and within hosts can play an important role in health and disease. To understand the capacity of skin bacteria to protect amphibian hosts from the fungal disease chytridiomycosis caused by *Batrachochytrium dendrobatidis* (Bd), we isolated 192 bacterial morphotypes from the skin of 28 host species of frogs (families Bufonidae, Centrolenidae, Hemiphractidae, Hylidae, Leptodactylidae, Strabomantidae, and Telmatobiidae) collected from the eastern slopes of the Peruvian Andes (540–3,865 m a.s.l.) in the Kosñipata Valley near Manu National Park, a site where we previously documented the collapse of montane frog communities following chytridiomycosis epizootics. We obtained isolates through agar culture from skin swabs of wild frogs, and identified bacterial isolates by comparing 16S rRNA sequences against the GenBank database using BLAST. We identified 178 bacterial strains of 38 genera, including 59 bacterial species not previously reported from any amphibian host. The most common bacterial isolates were species of *Pseudomonas, Paenibacillus, Chryseobacterium, Comamonas, Sphingobacterium*, and *Stenotrophomonas*. We assayed the anti-fungal abilities of 133 bacterial isolates from 26 frog species. To test whether cutaneous bacteria might inhibit growth of the fungal pathogen, we used a local Bd strain isolated from the mouthparts of stream-dwelling tadpoles (*Hypsiboas gladiator*, Hylidae). We quantified Bd-inhibition *in vitro* with co-culture assays. We found 20 bacterial isolates that inhibited Bd growth, including three isolates not previously known for such inhibitory abilities. Anti-Bd isolates occurred on aquatic and terrestrial breeding frogs across a wide range of elevations (560–3,695 m a.s.l.). The inhibitory ability of anti-Bd isolates varied considerably. The proportion of anti-Bd isolates was lowest at mid-elevations (6%), where amphibian declines have been steepest, and among hosts that are highly susceptible to chytridiomycosis (0–14%). Among non-susceptible species, two had the highest proportion of anti-Bd isolates (40 and 45%), but one common and non-susceptible species had a low proportion (13%). In conclusion, we show that anti-Bd bacteria are widely distributed elevationally and phylogenetically across frog species that have persisted in a region where chytridiomycosis emerged, caused a devastating epizootic and continues to infect amphibians.

## Introduction

Emerging infectious disease is a growing threat to global health and is identified as a major factor involved in the current biodiversity crisis (Daszak et al., 1999). Amphibians are considered one of the most threatened group of vertebrates on earth (Stuart et al., 2004; Wake and Vredenburg, 2008; Catenazzi, 2015), and the recently emerged disease chytridiomycosis, caused by the fungal pathogen *Batrachochytrium dendrobatidis* (Bd), has decimated species of amphibians in many parts of the world (Berger et al., 1998; Skerratt et al., 2007; Kilpatrick et al., 2010). The microbiota that live on and within hosts can play an important role in health and disease (Harris et al., 2009; Hanada et al., 2010; Hoyt et al., 2015; Ford and King, 2016). To understand the role of skin bacteria in protecting amphibians from chytridiomycosis, we document the distribution of amphibian skin bacteria with antifungal properties in host communities along the eastern slopes of the tropical Andes where amphibian populations have collapsed after Bd epizootics (Catenazzi et al., 2011). This region has the highest amphibian species richness on Earth (Hutter et al., 2017), and along our elevational gradient amphibian richness changes from more than 60 species in the Andean foothills to six species of amphibians in the high-elevation grasslands (Catenazzi et al., 2013). In our previous work (Burkart et al., 2017), we showed that culturable skin bacteria inhibiting Bd growth (henceforth, anti-Bd isolates) is linked to resistance to chytridiomycosis in high-elevation frogs. Here, we extend this approach to the broader elevational gradient, including elevations where species declines have been steepest from 1,250 to 1,750 m a.s.l. (Catenazzi et al., 2011), and relate the presence of anti-Bd bacteria on frogs to host susceptibility to chytridiomycosis (Catenazzi et al., 2017).

Amphibian skin provides an excellent environment for the growth of a wide variety of microorganisms (Kueneman et al., 2014; Bletz et al., 2017; Prado-Irwin et al., 2017). These microbial communities are functionally important for the host, for example in the defense against pathogens, such as Bd (Bettin and Greven, 1986; Harris et al., 2006; Belden and Harris, 2007; Culp et al., 2007; Woodhams et al., 2007b; Lauer et al., 2008; Becker and Harris, 2010; Flechas et al., 2012). Although many amphibian species have succumbed to epizootics of chytridiomycosis, others persist despite infection. Resistance and tolerance to chytridiomycosis has been attributed to the presence of beneficial bacteria in the skin, which can delay or inhibit the growth of the pathogen allowing host survival (Harris et al., 2006, 2009; Woodhams et al., 2007a).

Amphibian skin bacteria are likely to vary across hosts and elevation, as seen in recent studies of other Neotropical amphibian communities (Bresciano et al., 2015; Hughey et al., 2017). A putative source of amphibian skin bacteria, soil bacterial communities are known to change with elevation and slope aspect on mountains (Bryant et al., 2008). We hypothesize two main types of drivers affecting bacterial species composition at our study site, one related to environmental variation and the other to host behavior. We hypothesized that the environment (water, soil, leaf surface, etc.) is the main source of bacteria colonizing the skin of amphibians and that a species’ natural history (elevational range, aquatic vs. terrestrial reproduction) influences its encounters with bacterial sources. At our study site the main determinant of environmental change is elevation, which is correlated with changes in temperature, rainfall, oxygen availability, and UV radiation. Bacterial species richness in three habitats (the phyllosphere, the mineral and the organic soil) does not vary consistently along the elevational gradient, but bacterial community composition varies across these three habitats, and within each habitat, across elevation (Fierer et al., 2011; Nottingham et al., unpublished). Thus, it is possible that the species composition of bacterial communities on amphibian skin could vary with elevation.

**FIGURE 1:**
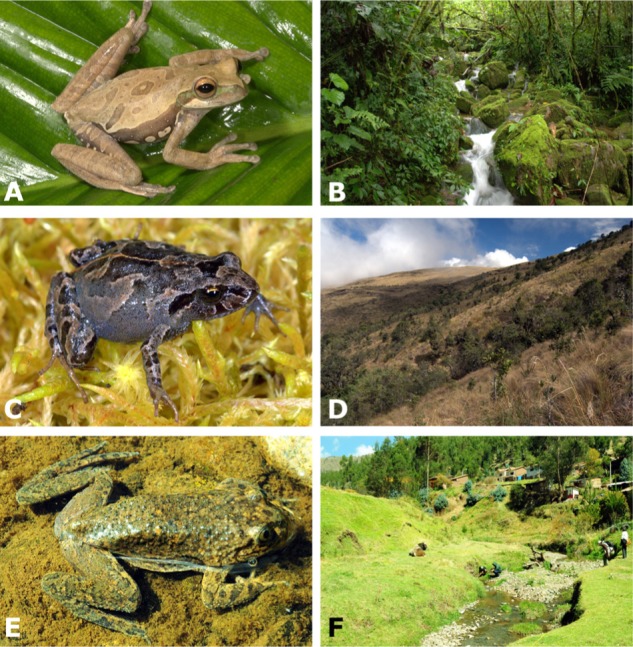
Representative amphibian hosts and their habitats sampled for this study: **(A,B)** *Hypsiboas gladiator* is non-susceptible to chytridiomycosis and lays aquatic eggs in streamside basins along montane streams in the cloud forest; **(C,D)** *Psychrophrynella usurpator* is non-susceptible and lays terrestrial eggs that undergo direct development under mosses in the high-Andean grassland; **(E,F)** *Telmatobius marmoratus* is highly susceptible to chytridiomycosis and lays aquatic eggs in small, high-Andean streams. Photographs by A. Catenazzi.

In addition to environmental factors, strains (or isolates; these two terms are interchangeable throughout the manuscript) colonizing the amphibian skin from the environmental pool may be filtered by host identity and behavior (McKenzie et al., 2012; Kueneman et al., 2014). Possible host-associated factors include skin morphology, the type and diversity of antimicrobial peptides secreted by skin, microhabitat use (e.g., use of microhabitats in close contact with soil; **Figure 1**), reproductive mode (e.g., requiring extended time in water), and hydric and thermoregulatory behaviors (Rollins-Smith and Woodhams, 2011; Bletz et al., 2017). Amphibians differ widely in their reproductive modes (Wells, 2007), which are linked to host affinity with aquatic environments. Adults of species with aquatic eggs and/or tadpoles often congregate in or around water bodies to attract mates, mate, lay eggs, and attend progeny (for species with parental care). In contrast, adults of species that lay eggs on land may rarely approach water bodies, and may be more homogeneously distributed on the forest litter or arboreal vegetation far from ponds or streams.

Traditional, culture-based methods to study bacterial diversity are limited in that only a fraction of the taxa present can be isolated and cultured. The use of next-generation sequencing has improved our understanding of microbial community composition (Qin et al., 2010; Fierer et al., 2011; Russo et al., 2012). Nonetheless, culture-based methods can provide accurate information on the functions of a specific bacterium (Becker et al., 2015b), and relevance of these functions to its symbiotic organism. Many studies have tested the antifungal properties of bacteria harbored on the skin of different amphibian species (Harris et al., 2006; Woodhams et al., 2007b; Lauer et al., 2008; Becker and Harris, 2010) and demonstrated that either a high proportion of anti-Bd bacteria, or bacteria with strong antifungal capacities, might explain the survival of some host species (Flechas et al., 2012; Burkart et al., 2017). Bacterial species belonging to the genera *Pseudomonas, Serratia*, and *Janthinobacterium* are among those that have demonstrated great abilities to inhibit Bd growth (Becker et al., 2015a,b; Bresciano et al., 2015), but there is a wide variety of other genera with similar abilities (Woodhams et al., 2015). Thus, there is a need to combine culture-based methods with molecular analyses to explore amphibian skin bacterial communities in relation to Bd resistance and tolerance (Lauer et al., 2008; Flechas et al., 2012; Muletz-Wolz et al., 2017).

We explored amphibian skin bacteria in host communities that experienced a collapse in species richness following epizootics of chytridiomycosis (Catenazzi et al., 2011, 2014). We previously discovered that the impact of Bd on amphibian communities was strongest among stream-breeding species (Catenazzi et al., 2011), and in amphibian communities at mid-elevations where Bd growth is optimal (Catenazzi et al., 2014). Many amphibians persist despite the continuous presence of Bd, but several of these persisting species continue to be susceptible to chytridiomycosis (Catenazzi et al., 2017). In this context, and considering how little is known about amphibian skin bacteria in the tropical Andes (Bresciano et al., 2015; Burkart et al., 2017), the overarching goal of this study is to examine the role of skin bacteria, and their anti-Bd abilities, in protecting amphibian hosts from disease at our study site. Here, we aim to (1) document species composition of bacterial isolates across elevation, host identity and behavior, (2) determine the inhibitory ability of bacterial isolates against the local strain of Bd, and (3) compare the distribution of anti-Bd isolates with respect to host susceptibility, host behavior and elevation. The wide elevational range, large number of hosts (28 total species), high susceptibility (six susceptible out of nine species with known status), and diversity of host reproductive modes (spanning from aquatic eggs and tadpoles to terrestrial viviparous embryos), provide an ideal opportunity to investigate the contribution of skin bacteria to host defense against chytridiomycosis.

## Materials and Methods

### Study System

We worked along the Paucartambo–Pillcopata road traversing the Kosñipata Valley and bordering Manu National Park and its buffer zone in southern Peru (Catenazzi et al., 2011, 2014). This road connects the high-Andean grasslands (puna) of the Cordillera de Paucartambo at the mountain pass of Acjanaco (3,400 m a.s.l.) to the Andean foothills (Pillcopata, 540 m a.s.l.) and Amazonian lowland rainforest of the upper Madre de Dios watershed. Owing to its wide elevational span and diversity of ecosystems, Manu NP protects the highest number of amphibian species of any protected area (Catenazzi et al., 2013). We sampled 131 frogs belonging to 28 species in seven families distributed from 560 to 3,865 m a.s.l. in the submontane and montane forests, montane scrub, and puna of the Kosñipata Valley (**Table 1**) during the dry season (14 June–22 August 2012). Furthermore, we include *Telmatobius marmoratus* (Telmatobiidae) obtained from the drier puna region surrounding the city of Cusco in June and July 2012. This species does not occur in the Kosñipata Valley, but is distributed on the western side of the Cordillera de Paucartambo at short distance from the Acjanaco mountain pass, and is known for having high Bd prevalence and susceptibility to Bd (Catenazzi et al., 2010, 2017; Warne et al., 2016).

**Table 1:** Species of anurans and elevations sampled for skin bacteria.

Host species (sample size)	#tes/isl	Elevation (m)	Reproduction [mode in Wells (2007)]
**Bufonidae**			
*Rhinella manu* (3)	0/4	1,920–2,100	Terrestrial, direct development (23)
**Centrolenidae**			
*Hyalinobatrachium bergeri* (3)	6/6	1,030–1,920	Epiphyllous eggs, lotic tadpoles (25)
**Hemiphractidae**			
*Gastrotheca antoniiochoai* (2)	2/2	2,915	Marsupial, direct development (35)
*Gastrotheca excubitor* (11)	14/15	3,340–3,695	Marsupial, direct development (35)
*Gastrotheca nebulanastes* (6)	9/9	2,790–2,920	Marsupial, direct development (35)
*Gastrotheca testudinea* (1)	2/2	2,100	Marsupial, direct development (35)
**Hylidae**			
*Dendropsophus rhodopeplus* (2)	2/2	560	Lentic eggs and tadpoles (1)
*Hypsiboas gladiator* (9)	10/12	1,350–1,450	Streamside basin eggs and tadpoles (4)
*Hypsiboas punctatus* (3)	2/3	560	Streamside basin eggs and tadpoles (4)
*Osteocephalus mimeticus* (1)	1/1	1,100	Streamside basin eggs and tadpoles (4)
*Scinax ruber* (4)	0/4	560–1,410	Lentic eggs and tadpoles (1)
**Leptodactylidae**			
*Adenomera andreae* (1)	1/1	540	Terrestrial foam nest, non-feeding tadpoles (32)
*Engystomops freibergi* (1)	1/1	540	Aquatic foam nest and lentic tadpoles (11)
**Strabomantidae**			
*Bryophryne cophites* (1)	2/2	3,865	Terrestrial, direct development (23)
*Noblella pygmaea* (1)	1/2	2,970	Terrestrial, direct development (23)
*Oreobates amarakaeri* (1)	1/2	560	Terrestrial, direct development (23)
*Pristimantis* cf. *cruciocularis* (1)	1/1	930	Terrestrial, direct development (23)
*Pristimantis danae* (10)	14/15	1,410–2,000	Terrestrial, direct development (23)
*Pristimantis* cf. *diadematus* (1)	1/1	1,065	Terrestrial, direct development (23)
*Pristimantis lindae* (1)	1/1	1,920	Terrestrial, direct development (23)
*Pristimantis pharangobates* (15)	3/28	1,920–2,790	Terrestrial, direct development (23)
*Pristimantis pluvialis* (1)	2/2	930	Terrestrial, direct development (23)
*Pristimantis* cf. *platydactylus* (6)	8/10	1,920–1,990	Terrestrial, direct development (23)
*Pristimantis salaputium* (10)	14/19	1,350–1,450	Terrestrial, direct development (23)
*Pristimantis* cf. *toftae* (7)	3/10	1,350–1,450	Terrestrial, direct development (23)
*Pristimantis* sp. (1)	1/1	2,350	Terrestrial, direct development (23)
*Psychophrynella usurpator* (13)	15/17	2,950–2,975	Terrestrial, direct development (23)
**Telmatobiidae**			
*Telmatobius marmoratus* (15)	16/19	3,400	Streamside basin eggs and tadpoles (4)
Total (sample size: 131)	133/192	540–3,865	

*^#^tes = number of morphotypes tested for Bd inhibition (duplicates considered as single morphotype); ^#^isl = number of morphotypes isolated from host species (after discarding duplicates). Note that not all morphotypes could be sequenced.*

### Ethics Statement

This work has been approved by the Animal Care and Use Committees of San Francisco State University (Protocol #A12-07) and Southern Illinois University (Protocol #13-027), and by the Peruvian Ministry of Agriculture. The Asociación para la Conservación de la Cuenca Amazónica authorized work at its Wayqecha Biological Station.

### Disease Prevalence

We estimated disease prevalence by swabbing 543 frogs captured from 540 to 3,865 m a.s.l. with sterile synthetic rayon swabs (MW113, Medical Wire and Equipment, England). We gently stroked the sterile swab across the skin of each frog a total of 30 times: five strokes on each side of the abdominal midline, five strokes on the inner thighs of each hind leg, and five strokes on the foot webbing of each hind leg (Catenazzi et al., 2011, 2017). We extracted DNA from swabs with Prepman Ultra (Life Technologies, Carlsbad, CA, United States). Each single swab extract was amplified once following a standard, probe-based quantitative Polymerase Chain Reaction protocol (Boyle et al., 2004; Hyatt et al., 2007) using a 7300 Real-Time PCR System (Life Technologies, Carlsbad, CA, United States), as reported in Catenazzi et al. (2017). This qPCR assay estimates Bd genomic equivalents (GEs) in each sample, and we converted GE values to provide “zoospore equivalents” on each frog by using genomic standards of known zoospore concentrations. We considered frogs to be infected if zoospore equivalents > 0, and non-infected if zoospore equivalents = 0. We calculated Bd prevalence (proportion of swabbed frogs infected with Bd) using Bayesian inference with Jeffrey’s non-informative priors with the ‘binom’ package in R (Dorai-Raj, 2014). Bd prevalence data are available online at the Amphibian Disease database^1^ at the URL: https://n2t.net/ark:/21547/AXY2.

### Bacterial and Bd Isolation

We obtained bacteria directly from skin swabs of frogs. We handled each frog with new nitrile gloves and rinsed them with distilled water prior to swabbing in order to remove transient cutaneous bacteria (Lauer et al., 2007). We sampled bacteria by running a sterile MW113 swab (not the same swab used for determining Bd infection) on the frog’s left and right sides and ventral surfaces, hindlimbs, and interdigital membranes for a total of 50 strokes (Flechas et al., 2012). We then streaked the swab on a petri dish of nutrient agar and incubated the dish at room temperature (14–18°C) until observing bacterial growth (4–6 days). We defined bacterial morphotypes according to macroscopic characteristics (color, form, elevation, and margin), and transferred each to fresh nutritive agar plates until pure cultures were obtained. We transported each isolate to the laboratory for Bd growth inhibition assays and identification through sequencing of the 16S rRNA gene (Weisburg et al., 1991).

We isolated a local strain of Bd from the mouthparts of *Hypsiboas gladiator* tadpoles collected in creeks of the Kosñipata Valley at 1,350 m a.s.l. We examined the mouthparts for signs of Bd infection, such as depigmentation and missing tooth rows (Fellers et al., 2001; Marantelli et al., 2004; Rachowicz and Vredenburg, 2004). We euthanized individuals with signs of disease by decapitation, their mouthparts dissected and cut into approximately 2 mm × 2 mm squares. We cleaned each piece of mouthpart in water-based agar with antibiotics in order to remove bacteria, yeast, and fungal spores. We then transferred the clean mouthparts to a fresh plate with TGh media (10 g tryptone, 10 g agar, 4 g gelatin hydrolysate, 1,000 mL distilled water). We transferred Bd colonies to fresh plates as soon as they appeared, and these were transported to the laboratory and maintained in culture on TGh agar at 23°C. We cryopreserved the Bd strain following standard procedure (Boyle et al., 2003).

### Bacterial DNA Extraction, Sequencing, and Identification

We swabbed agar plates with pure cultures of each bacterial strain, then extracted DNA from these swabs using Prepman Ultra, and amplified DNA by PCR with GoTaq Green Master Mix (Promega Corporation, Madison, WI, United States) and 16S primers 27F and 1492R (Lane, 1991). After verifying PCR products by gel electrophoresis, we sequenced each isolate’s DNA (MCLAB, San Francisco, CA, United States). We aligned sequences (only those with >60% high quality base pairs) using Geneious v8.1.9 (Biomatters Limited, Auckland, New Zealand), and compared consensus sequences to microbial DNA sequences on the NCBI database (using BLAST default parameters). Isolates that were >99% similar were considered a match (Burkart et al., 2017).

We could not assay 50 of our bacterial isolates for Bd inhibition (see below). Therefore, we attempted to infer inhibitory status for these isolates from a published dataset (Woodhams et al., 2015). We also included in this comparison our assayed isolates to examine consistency of inhibitory status across studies. In order to compare our sequenced isolates with the published literature, we retained 159 sequences after discarding five duplicate and three triplicate sequences and 19 low quality reads. These 159 sequences (see GenBank accession codes in Supplementary Table S1) were added to the dataset of amphibian skin bacteria of Woodhams et al. (2015), and the combined list of 2102 strains was aligned using MAFFT v7.0 using the default option FFT-NS-2 (Katoh et al., 2017). The online interface we used^2^ allows computation of multiple sequence alignment for large datasets (i.e., thousands of sequences), and calculates genetic distances for the aligned sequences. We used 1% 16S genetic distance as our criterion for delimitation of operative taxonomic units (OTUs).

### Bd Growth Inhibition Assays

We used agar plate co-cultures to assay the ability of bacterial strains to inhibit Bd growth. We flushed plates with a Bd-enriched broth to produce a homogeneous distribution of Bd colonies. Then we streaked a line of the bacterial isolate on one side of the plate, parallel to a second line of a bacterial strain known to lack anti-Bd properties (*Escherichia coli* strain DH5α) serving as negative control. We assayed bacterial strains in triplicate. After 3 days incubated at 23°C, we visually inspected plates and classified the bacterial strains as either non-inhibitory (no zone of Bd growth inhibition present) or potentially inhibitory (clear zone of Bd growth inhibition present). We examined plates after 3 days because that time is when maximum Bd zoospore production was observed during our protocol optimization procedures. Furthermore, many bacteria grew quickly, and the third day was optimal for quantifying Bd inhibition and comparing values among bacterial isolates.

We quantified the relative distance from the streak of the query bacterium to where 50% of maximum Bd growth occurred, and used this value to compare each isolate’s strength at inhibiting Bd (Burkart et al., 2017). Isolates with stronger inhibitory abilities depressed Bd growth over longer distances. We recorded Bd growth by measuring the gray values of standardized photographs of the petri dishes using ImageJ software (Flechas et al., 2012; Burkart et al., 2017). Gray values (hereafter referred to as “growth”) are measurements of the intensity of light in a pixel of a black and white image, and by averaging among pixel wide columns across the area between the query bacterium and negative control, we quantified growth over minute distance increments from the query. Distances between the query and the negative control varied slightly between petri dishes, so we expressed them as percentages of the total length (i.e., relative distance) to facilitate comparative analysis. We expressed growth as percentage of the maximum growth recorded to control for any variation between petri dishes. For each replicate, we plotted values on a graph using Microsoft Excel (Microsoft Corporation, Redmond, WA, United States), and determined the distance at which 50% growth occurred using the function of a line fit to the graphed values (Burkart et al., 2017). We report average relative distance and standard error calculated across the three replicates of each isolate.

Four bacterial strains were assayed twice (because strains had not been sequenced prior to assays, and thus morphologically variable strains were thought to be distinct following visual examination), three strains were non-inhibitory across the two trials (*Paenibacillus* sp. 2; *Pseudomonas* sp. 24; *Chryseobacterium lactis*); whereas *Pseudomonas* sp. 2 was inhibitory in the first trial (average inhibition strength of 67%), and non-inhibitory in the second trial. These four strains were considered to be non-inhibitory for our analyses.

### Host Susceptibility Data and Statistical Analyses

We used data from infection experiments conducted as part of another study (Catenazzi et al., 2017) to relate host susceptibility to proportion and strength of anti-Bd bacterial strains. Frogs of eight species sampled in this study (*Gastrotheca excubitor, G. nebulanastes, Hypsiboas gladiator, Pristimantis danae, P.* cf. *platydactylus, P. pharangobates, P. toftae, Psychrophrynella usurpator*) were exposed to highly infected individuals of *Telmatobius marmoratus*. Following infection, we compared survival between groups of Bd-exposed and non-exposed (control) individuals (duration of experiments varied by species). Frogs were cleared of Bd infection before the experiments by immersion in a 1% itraconazole solution for 5 min a day for seven consecutive days. We used Cox’s proportional hazards model with censoring to assess the risk of dying for Bd-exposed frogs (Catenazzi et al., 2017). Here, we use the predicted days to death from Cox’s proportional hazards models for each species as reported in Catenazzi et al. (2017), and relate this value to the proportion and inhibitory strength of anti-Bd strains. We used analysis of variance to determine whether the proportion of anti-Bd isolates predicts the number of days to death. For descriptive purposes, we consider host species to be Bd-susceptible whenever our survival analysis models in Catenazzi et al. (2017) produced an estimate for number of days to death. We also include data of survival for *T. marmoratus* (not included in Catenazzi et al., 2017) comparing survival between infected and treated frogs (immersion in itraconazole baths).

We examined patterns of bacterial composition at two taxonomic levels: strain (OTU) and phylum. We used analysis of variance to test the significance of linear regression relating frog sample size with number of OTUs. In order to explore the effect of elevation on number of OTUs, skin bacterial composition and proportion of anti-Bd isolates, we used analysis of variance and pooled samples into seven 500 m elevational classes (540–999 m, 1,000–1,499 m, etc. up to 3,500–3,865 m a.s.l.). We also fitted a polynomial, quadratic curve to model the relationship between elevation and the proportion of anti-Bd isolates. In order to explore the effect of host reproductive behavior, we used a χ^2^ contingency table test with the categories of reproductive mode proposed by Wells (2007), arranged along a continuum from aquatic eggs and tadpoles, to direct development on land, and viviparity (marsupial frogs), as listed in **Table 1**. For dimensional analyses of skin bacterial communities we used non-metric multidimensional scaling (NMDS). NMDS produces an ordination, i.e., conflates information from multiple dimensions into a few dimensions based on a distance or dissimilarity matrix. NMDS is usually considered as a more robust and flexible technique than other ordination techniques because the original distances are replaced with ranks (Minchin, 1987). While the information on distance magnitude is thus lost, the rank based approach is more robust for data lacking an identifiable distribution. Furthermore, mixed quantitative and qualitative variables can be used. Because there was little overlap of bacterial species and genera among amphibian hosts, we considered data on distribution of phyla for NMDS. We performed analyses in the R package vegan (Oksanen et al., 2015) by using the “metaMDS” function for NMDS, the “rankindex” function to determine the best method for calculating the distance matrix from our data, and the “adonis” function to perform analysis of variance (permutation test with pseudo-F ratios) using the distance matrices and five categorical variables (elevation, reproductive mode, host sample size, and host susceptibility). For the purpose of these analyses of variance, we conflated reproductive modes into two categories (terrestrial eggs and aquatic eggs), and performed the analysis for host susceptibility on a reduced dataset (information on susceptibility only available for nine of the 28 sampled amphibian species). We report averages ± SE.

## Results

### Composition of Cutaneous Bacteria

We isolated 199 bacterial morphotypes from the skin of 131 frogs distributed across 28 species and seven anuran families, and from 540 to 3,865 m a.s.l. (**Table 1**). We were able to sequence 185 of these 199 morphotypes, and among these 185 sequences, we identified five duplicates and one triplicate, reducing the number of bacterial isolates to 178 OTUs (Supplementary Table S1). These OTUs were distributed across four bacterial phyla: Proteobacteria, Actinobacteria, Firmicutes, and Bacteroidetes.

**FIGURE 2:**
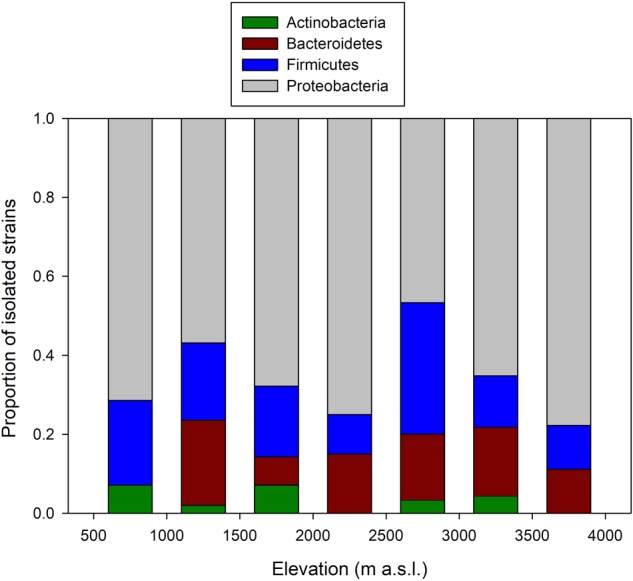
Change in the composition of frog skin bacterial isolates arranged by phyla along the elevational gradient in the Kosñipata Valley near Manu National Park, SE Peru.

The number of OTUs varied with host sample size (*F*_1,26_ = 366.3, *p* < 0.001), and after taking into account this sampling effect, there was no change in the average number of OTUs per host across elevation (*F*_1,5_ < 0.01, *p* = 0.977). There were some changes in the relative proportion of phyla across elevation (**Figure 2**). At all elevations, isolated skin bacteria were dominated by Proteobacteria, followed by Firmicutes, Bacteroidetes, and Actinobacteria, but the relative proportion of Bacteroidetes peaked at 1,000–1,499 m a.s.l., and that of Firmicutes at 2,500–2,999 m a.s.l.

Analyses of variance (permutation test) showed that host sample size (*F*_1,23_ = 51.29; *p* = 0.001) and elevation (*F*_1,23_ = 4.13; *p* = 0.034) significantly affected phyla community composition across amphibian hosts (Supplementary Figure S1), but there were no effects of egg laying site (*F*_1,23_ = 0.67; *p* = 0.521), and host susceptibility (*F*_1,7_ = -0.08; *p* = 0.978).

### Distribution of Anti-Bd Bacteria and Host Susceptibility to Chytridiomycosis

We assayed 137 morphotypes, belonging to 133 isolates (four morphotypes were assayed twice), sampled from 26 species of frogs. Six of the 133 strains could not be sequenced (“undetermined bacteria”; Supplementary Table S1), five of which were inhibitory. Therefore, our dataset of assayed strains includes 126 OTUs identified through sequencing of 16S rRNA, and six unidentified isolates.

We found 21 anti-Bd bacterial isolates in nine host species of six genera (*Dendropsophus, Gastrotheca, Hypsiboas, Pristimantis, Psychrophrynella*, and *Telmatobius*; **Table 2**), with only two species, *G. excubitor* and *H. gladiator*, having more than four anti-Bd isolates each. These hosts and their associated anti-Bd bacteria live at elevations from 560 to 3,695 m a.s.l., in habitats that span from foothill tropical rainforest to cloud forest and high-Andean grassland, and hosts that lay eggs in ponds (*Dendropsophus*), streams (*Hypsiboas, Telmatobius*), moist terrestrial environments (*Pristimantis, Psychrophrynella*), and that retain eggs in specialized dorsal pouches (*Gastrotheca*).

**Table 2:** Distribution and inhibitory strength (as measured by the relative distance from the streak of the query bacterium to point of 50% of max Bd growth; see Materials and Methods) of anti-Bd bacterial isolates across host species.

Host species and isolate	Elevation (m)	Distance	SE
***Dendropsophus rhodopeplus***			
*Pseudomonas entomophila*	560	78.04%	2.20%
*Serratia marcescens*	560	42.43%	14.97%
***Gastrotheca excubitor***			
*Pseudomonas sp. 2*	3,340	67.21%	10.39%
*Pseudomonas sp. 34*	3,340	61.44%	10.77%
*Pseudomonas sp. 18*	3,340	58.80%	7.67%
*Rahnella aquatilis*	3,695	50.25%	9.10%
*Pseudomonas sp. 1*	3,695	47.91%	1.91%
*Janthinobacterium lividum*	3,695	32.86%	3.08%
***Gastrotheca nebulanastes***			
*Pseudomonas sp. 25*	2,850	38.44%	N/A
***Hypsiboas gladiator***			
*Undetermined bacterium 14*	1,410	69.98%	6.85%
*Undetermined bacterium 13*	1,410	61.38%	1.76%
*Rahnella sp. 2*	1,410	56.37%	4.47%
*Undetermined bacterium 1*	1,450	49.69%	9.77%
*Sphingobacterium faecium*	1,350	43.68%	N/A
***Pristimantis cf. diadematus***			
*Paenibacillus sp. 24*	1,065	66.64%	1.29%
***Pristimantis danae***			
*Pseudomonas sp. 36*	1,920	58.94%	9.96%
*Paenibacillus sp. 3*	2,000	45.73%	6.93%
***Pristimantis salaputium***			
*Undetermined bacterium 2*	1,350	73.50%	10.48%
***Psychrophrynella usurpator***			
*Undetermined bacterium 5*	2,970	58.26%	9.31%
*Pseudomonas azotoformans*	2,975	51.11%	11.02%
***Telmatobius marmoratus***			
*Pseudomonas fluorescens*	3,400	41.77%	2.43%

*Bacteria are ranked according to their inhibitory abilities.*

**FIGURE 3:**
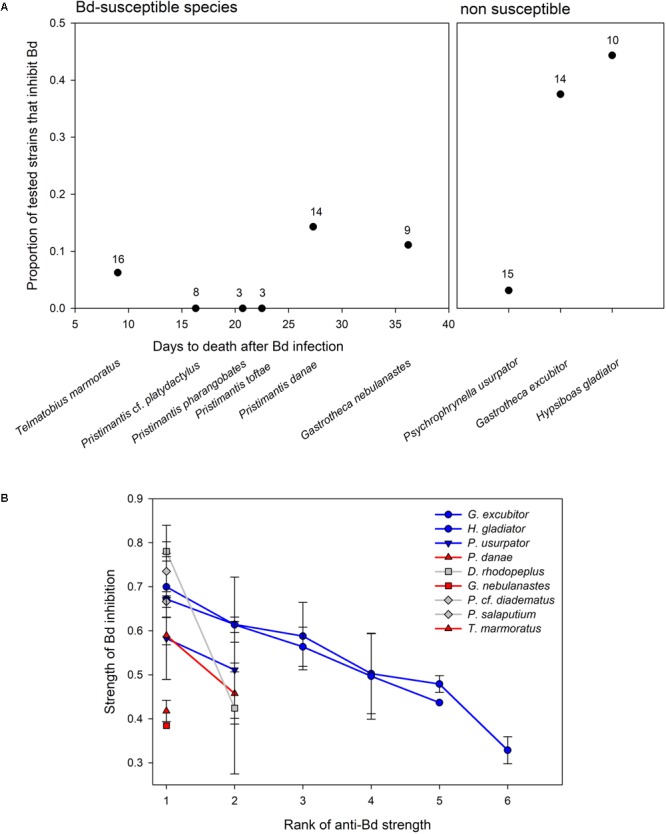
**(A)** Variation in proportion of anti-Bd bacterial isolates in Bd-susceptible and non-susceptible amphibian hosts. In susceptible species, days to death after Bd infection are calculated from survival analysis (Catenazzi et al., 2017). Number of tested bacterial morphotypes reported above each data point. **(B)** Average strength (±SE) of Bd inhibition of anti-Bd isolates (as measured by the relative distance from the streak of the query bacterium to point of 50% of max Bd growth; see Materials and Methods) ranked by inhibitory strength (from strongest to weakest inhibitor) in three Bd-susceptible hosts (in red), three non-susceptible hosts (in blue), and three hosts of unknown Bd susceptibility (i.e., not tested, in gray). Bacterial strains vary by frog host species.

The proportion of anti-Bd isolates ranged from 0 to 14% in six host species that were susceptible to Bd, but there was no relationship between number of days to death and proportion of anti-Bd isolates (*F*_1,4_ = 1.46, *p* = 0.293; **Figure 3A**). Among the three non-susceptible host species, *G. excubitor* and *H. gladiator* had the largest proportions of anti-Bd isolates (40 and 45%, respectively), but *P. usurpator* only had 13% anti-Bd isolates, within the range of Bd-susceptible hosts.

The strength of Bd inhibition averaged 55.0 ± 2.6% across all isolates, and ranged from 32.9 to 78.0% (**Table 2** and **Figure 3B**). Although data points are too few to compute statistical analyses, the inhibition strengths of anti-Bd isolates were generally higher in non-susceptible hosts than they were in Bd-susceptible hosts, with the exception of *P. usurpator*. Only three of the six Bd-susceptible hosts are shown in **Figure 3B**, because the other three species had no anti-Bd isolates. Hosts of unknown susceptibility to Bd had anti-Bd isolates with inhibition strength similar to that of non-susceptible species.

**FIGURE 4:**
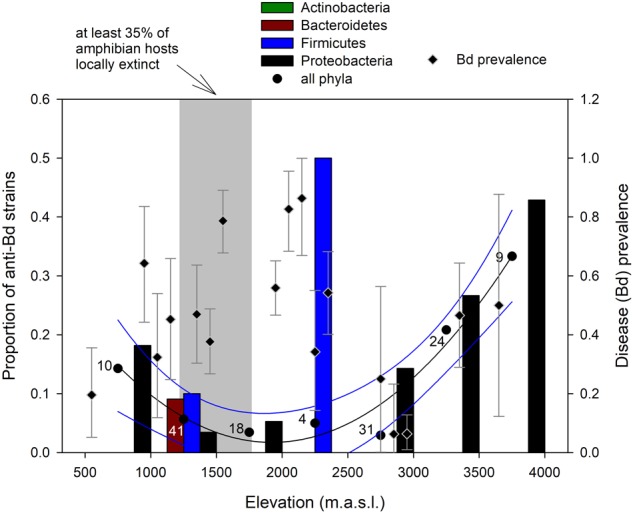
Change in proportion of anti-Bd bacterial strains within phyla (bars) and across all phyla (circles; numbers indicate host sample size) along the elevational gradient in the Kosñipata Valley near Manu National Park, SE Peru. Diamonds indicate disease (Bd) prevalence with Bayes credible intervals in amphibian hosts, and the shaded area indicates elevations at which at least 35% of amphibian species have disappeared following the Bd epizootics (Catenazzi et al., 2011).

The proportion of anti-Bd isolates varied with elevation (*F*_2,4_ = 36.05, *p* = 0.0028; **Figure 4**), following a polynomial, quadratic curve that reaches its lowest values at mid-elevations from 1,500 to 2,000 m a.s.l., partially overlapping with elevations from 1,250 to 1,750 m a.s.l. where at least 35% of amphibian hosts became extinct during the Bd epizootics (Catenazzi et al., 2011). These elevational ranges also partially overlapped with the 1,500–2,200 m a.s.l. range where prevalence of Bd infection was highest during our sampling period in the dry season of 2012 (Supplementary Table S2).

**FIGURE 5:**
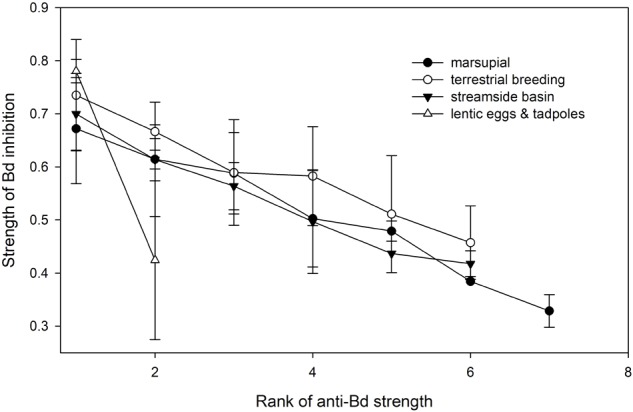
Variation in average strength (±SE) of Bd inhibition of anti-Bd isolates (ranked from strongest to weakest inhibitor) across host reproductive modes.

Anti-Bd isolates occurred across four types of reproductive modes (**Figure 5**): aquatic eggs and tadpoles in ponds, aquatic eggs and tadpoles in streamside basins, terrestrial breeding with direct development, and marsupial brooding with direct development. There was no effect of reproductive mode on the proportion of anti-Bd isolates (χ^2^ = 10.372, df = 6, *p* = 0.110) and, among the four reproductive modes with anti-Bd isolates, no effect on the inhibitory strength of anti-Bd isolates (*F*_3,46_ = 0.68, *p* = 0.571; **Figure 5**).

### Comparison With Published Literature

The alignment produced through MAFFT v7.0 generated shorter genetic distances than those calculated for our isolates in Geneious. Despite these shorter distances, 59 of our 159 sequenced isolates had no matches (our OTUs defined as those having 16S genetic distance <1%) among the 1,944 previously published isolates listed in Woodhams et al. (2015). The remaining 100 isolates produced 445 matches with isolates in the published dataset, with some isolates of *Pseudomonas* generating up to 100 (*Pseudomonas entomophila*), 81 (*Pseudomonas* sp. 7), and 74 matches (*Pseudomonas* sp. 2 and *Pseudomonas* sp. 24). There was little consistency in inhibitory status among OTUs. None of the OTUs we identified as inhibitory were inhibitory across all studies summarized in the published dataset. Likewise, none of the OTUs we identified as non-inhibitory were non-inhibitory across all studies summarized in the published dataset. Therefore, we decided not to use OTU as a predictor of inhibitory status for the isolates that we could not assay against Bd.

## Discussion

We show that bacterial isolates inhibiting the fungal disease chytridiomycosis are distributed across a wide range of environmental conditions (temperature and rainfall, as they vary along elevation), host families and genera, host susceptibility status, and microhabitat associated with host reproductive behavior. Our findings suggest anti-Bd isolates may play a role in host defense against chytridiomycosis, maintenance of host community composition, and the elevational pattern of Bd infection prevalence. We found the highest proportion and inhibition strengths of anti-Bd isolates in two non-susceptible species, while Bd-susceptible species had low proportions (<15%) of weakly inhibitory bacterial isolates. Intriguingly, we also recorded low proportion, and weak inhibitory strength in a common, non-susceptible species. Our attempt at inferring inhibitory status of bacterial isolates by matching their 16S sequences with sequences from previous studies was unsuccessful, suggesting that OTU identity is a poor predictor of inhibition strength.

Our findings acquire relevance in the context of our study system, which consists of species-rich amphibian communities that have been decimated by chytridiomycosis. This highly virulent disease infects the skin and interferes with the organ’s functions, eventually leading to death in many amphibian species (Voyles et al., 2009; Kilpatrick et al., 2010). We have previously documented the collapse of montane forest amphibian communities at our study site (Catenazzi et al., 2011, 2014), following epizootics of chytridiomycosis that reached southern Peru in the early 2000s (Catenazzi and von May, 2014). During these epizootics nearly 20 out of 60 species of amphibians were extirpated from the Kosñipata Valley, and many more declined in abundance or disappeared from part of their previous elevational range. While the host/Bd dynamics have shifted from epizootic to enzootic (Catenazzi et al., 2017), and communities of surviving species do not seem to undergo further declines, we recently demonstrated that many surviving host taxa continue to be susceptible to chytridiomycosis (Catenazzi et al., 2017). Therefore, host-specific factors, or the dynamics of the enzootic host/Bd system, currently prevent Bd from causing widespread host mortality and further population declines. With this study we have explored one such factor, host skin defense provided by symbiotic bacteria.

Our primary interest was to examine any relationship between distribution of anti-Bd isolates and host susceptibility in the context of amphibian communities ravaged by chytridiomycosis. We compared the elevational distribution of anti-Bd isolates with the elevational range corresponding to the sharpest declines in amphibian diversity (where at least 35% of frog species have been extirpated), and with elevational variation in Bd infection prevalence. Difference in elevational resolution among these datasets prevented more rigorous statistical analyses; data on proportion of declining amphibian species and Bd prevalence are available for 100 m elevational classes (Catenazzi et al., 2011), whereas data on anti-Bd isolates were pooled into 500 m elevational classes to minimize differences in sample size among classes. Assuming the observed patterns are representative, our data suggest that the lowest proportion of anti-Bd isolates overlap with regions of high Bd prevalence and high host extirpation, which creates a conundrum. One would expect the opposite pattern to occur if Bd was selecting for host resistance. If symbiotic bacteria were contributing to skin defenses, then hosts that survived Bd epizootics in disease-ravaged communities should have greater proportions of anti-Bd bacteria than survivors in communities that had not been strongly diminished by disease. Another possibility is that most frogs at mid-elevations lacked abundant beneficial symbionts, explaining why so many species were extirpated during epizootics, and that surviving species continue to lack symbionts, but are able to persist for other reasons. Other skin defenses, such as antimicrobial peptides, play important roles in inhibiting Bd growth and delaying the lethal effects of chytridiomycosis (Rollins-Smith et al., 2002; Melzer and Bishop, 2010; Holden et al., 2015). Moreover, diminished frog density and lower number of host species following epizootic outbreaks, as well as the transition of the host/Bd dynamics from epizootic to enzootic likely reduces rates of transmission and the availability of environmental disease reservoirs (Briggs et al., 2010), allowing host species to persist and even recover (Knapp et al., 2016).

An alternative hypothesis to the conundrum posed by the U-shaped curve in elevational distribution of anti-Bd isolates is that high prevalence of Bd, and thus high probability of infection in hosts, is disrupting skin bacterial communities (Jani et al., 2017), and disproportionally affecting anti-Bd bacteria relative to co-occurring bacteria. This alternative hypothesis would again imply that other factors, unrelated to skin symbionts, are preventing high Bd virulence in these hosts. Continuous exposure to Bd in regions of high prevalence, for example, could strengthen acquired immunity in hosts (McMahon et al., 2014), although our experimental data (Catenazzi et al., 2017) and findings from other studies show limited support for immunoprotective effects in many species of amphibians (Stice and Briggs, 2010; Cashins et al., 2013; Hudson et al., 2016; Geiger et al., 2017).

Support for a role of symbiotic bacteria in frog skin defense is provided through our comparison of the inhibitory strength and proportion of anti-Bd isolates in hosts that were experimentally infected with Bd. With the exception of one common and non-declining frog species (*P. usurpator*), we found that non-susceptible species had the highest proportion of strongly inhibitory anti-Bd isolates, whereas Bd-susceptible species had a low proportion of weakly inhibitory anti-Bd isolates. We previously associated variation in the host response to Bd infection in marsupial frogs to the presence of bacteria with antifungal properties (Burkart et al., 2017). We confirmed this association along the broader elevational gradient and across nine frog species from four families. For the case of *P. usurpator*, a species that persisted and even increased in abundance despite the presence of Bd (Catenazzi et al., 2017), the proportion of anti-Bd bacteria is very low and similar to what we found for Bd-susceptible species, and for species found at mid-elevations. Bd prevalence and infection loads in *P. usurpator* are generally lower than in sympatric frog species (Catenazzi et al., 2011, 2017), suggesting that this species might avoid infection, or that its skin might excel at preventing zoospore penetration, or that its adaptive immune system is more effective at resisting chytridiomycosis (Savage and Zamudio, 2011). This frog breeds terrestrially and does not use aquatic habitats where Bd zoospores occur at highest densities, nor does it congregate for reproduction, thus limiting potential for Bd transmission from both environmental reservoirs and highly infected individuals. Behavioral effects and independence from water bodies may also explain why other terrestrial-breeding species, such as *P. toftae* which occurs at mid-elevation in regions of high Bd prevalence, can continue to persist without protection from anti-Bd bacteria.

Comparisons of our strains with a published dataset, after alignment of 16S sequences and using 1% genetic distance as criterion for OTU delimitation, revealed that OTU identity is a poor predictor of inhibition status and strength, to the extent that it thwarted our attempt to determine inhibition strength for isolates that we could not assay against Bd. Although bacterial genera such as *Jantinobacterium, Serratia, Stenotrophomonas, Aeromonas*, and *Pseudomonas* are frequently associated with anti-Bd capacity (Harris et al., 2009; Becker et al., 2015b; Woodhams et al., 2015), emergent traits of bacterial communities, including community structure and species richness (Woodhams et al., 2014; Becker et al., 2015a; Piovia-Scott et al., 2017), might be more predictive of host resistance to chytridiomycosis than data from co-culture assay testing single bacterial isolates. These findings are important to inform probiotic treatments aimed at mitigating the effects of chytridiomycosis on amphibian hosts (Bletz et al., 2013; Kueneman et al., 2016).

Our investigation, which was performed by using culturable bacteria, may provide a limited representation of overall bacterial diversity yet has the advantage of producing isolates that can be tested for anti-Bd capacity (Becker et al., 2015b). Previous work has demonstrated that most dominant skin bacteria can be isolated and cultured (Walke et al., 2015), so the undetected bacteria may not constitute an extensive group. Furthermore, culturable isolates were needed to determine isolate-specific inhibition strength against the local strain of Bd. These strain-specific inhibition data were especially informative because we previously determined host susceptibility to the same local Bd strain *in situ* (Catenazzi et al., 2017), and because inhibitory capacity of bacteria appears to vary when different Bd strains are assayed (Antwis et al., 2015). By sampling bacteria directly from wild frogs, testing them against a local strain of Bd, and relating our findings to known patterns of disease prevalence and susceptibility, we aim to minimize external factors that would interfere with our interpretation regarding the role of symbiotic bacteria as a line of defense against fungal disease.

The environment has been hypothesized as the main source of symbiotic bacteria in frogs (Muletz et al., 2012), although there is evidence that the core of host bacterial communities may occur independently of environment (Wiggins et al., 2011; Loudon et al., 2014). Vertical transmission of bacteria to offspring has also been suggested as an alternative route for species with parental care (Walke et al., 2011; Burkart et al., 2017). Among our sampled species, four species of *Gastrotheca* have direct development with embryos enclosed in a sealed dorsal pouch until hatching, when they emerge through the opening of the brooding pouch. The pouch is made of modified skin, and even allows for nutrient provisioning by the brooding mother (Warne and Catenazzi, 2016). It is reasonable to imagine that such intimate contact between mother and progeny promotes vertical transmission of symbiotic bacteria at birth (Bresciano et al., 2015; Burkart et al., 2017). In another sampled species, *Hyalinobatrachium bergeri*, males attend nests of epiphyllous eggs, and could transfer skin microbes to developing embryos (Walke et al., 2011). Despite this natural history, which might provide an explanation for differences in the relative proportion of core, vertically transmitted and possibly transient, environmentally acquired bacteria, our analyses at the coarse level of phyla did not detect any effect of host reproductive mode. A more comprehensive characterization of bacterial diversity using next-generation sequencing will improve examination of sources of bacterial symbionts in our amphibian species.

Host reproductive mode might also affect skin bacterial composition because amphibians breed in a variety of aquatic, terrestrial and arboreal environments, thus exposing the skin to different environmental source pools of microbes. Although our coarse, phylum level analyses did not reveal any variation in skin bacterial composition, nor variation in the proportion and strength of anti-Bd isolates among host reproductive modes, it is possible that more comprehensive and finer analyses of bacterial diversity (i.e., at the species level) may detect an effect of host behavior. Furthermore, future research that quantifies relative abundances of the different symbiotic bacteria, for example by using count plates (Bresciano et al., 2015) or quantitative PCR (Piovia-Scott et al., 2017) from skin swab samples, would help us understand the complexity of bacterial communities on amphibian skin.

Among environmental factors influencing bacterial diversity, elevation can be used as a proxy for temperature, which decreases at a rate of ∼0.55°C every 100 m in elevation, and for rainfall, which ranges from over 4 m at the foothill of the Andes to less than 1 m on the western side of the Cordillera de Paucartambo (Catenazzi et al., 2014). These changes in temperature and rainfall may act directly on soil, rock and leaf bacteria living in the environment, or indirectly on skin symbionts through the body temperature of amphibian hosts. Furthermore, ecological interactions among skin symbionts, fungal pathogen, and other symbiotic microbes and parasites may influence bacterial composition along the elevational gradient. The patterns of cutaneous bacterial diversity, as well as their role in skin defense, likely result from the interplay of multiple forces: temperature and moisture effects on pathogen (Piotrowski et al., 2004; Stevenson et al., 2013), temperature and moisture effects on host (Raffel et al., 2006, 2015; Catenazzi et al., 2014) and bacteria (Fierer et al., 2011; Nottingham et al., unpublished), and ecological interactions among co-occurring microbes (Woodhams et al., 2014; Piovia-Scott et al., 2017).

## Conclusion

We found that anti-Bd bacteria are widely distributed across bacterial phyla and genera, occur along a wide elevational range in the Amazon to Andes transition, and are found on amphibian hosts that use aquatic, terrestrial and arboreal environments. The pattern of elevational distribution of anti-Bd isolates, and the association of high proportion of anti-Bd isolates of high inhibitory strength with low host susceptibility to disease, support the idea that symbiotic bacteria play a functional role in amphibian skin defense. Yet this association does not consistently explain the fate of amphibian hosts along the elevational gradient, suggesting complex interactions among bacterial symbionts, hosts, and environmental factors in determining frog persistence in a region of high disease prevalence.

## Author Contributions

AC, SVF, and VTV designed the study, developed the methodology, and collected the field data. AC and VTV acquired the funding. AC, SVF, DB, NH, and JT conducted the lab work. AC supervised the data curation and conducted the analyses. AC and SVF wrote the original draft. All authors reviewed and edited drafts.

## Conflict of Interest Statement

The authors declare that the research was conducted in the absence of any commercial or financial relationships that could be construed as a potential conflict of interest.

## Footnotes

1 https://amphibiandisease.org/

2 https://mafft.cbrc.jp/alignment/server/
