# Supplementary material for: Widespread Elevational Occurrence of Antifungal Bacteria in Andean Amphibians Decimated by Disease: A Complex Role for Skin Symbionts in Defense Against Chytridiomycosis
Source: Front Microbiol. 2018 Mar 14;9:465. doi: 10.3389/fmicb.2018.00465 (PMC5861192; doi:10.3389/fmicb.2018.00465)
Supplement: Supplementary file 1 [file Table_1.DOCX]

**Table S1. List of bacterial strains and accession numbers.**

| **Host and bacterial species** | **Elevation (m)** | **#isolates** | **Tested against Bd** | **Anti-Bd strains** | **Genbank accession** |
| --- | --- | --- | --- | --- | --- |
|  |  |  |  |  |  |
| ***Bryophryne cophites*** | **3865** | **2** | **2** |  |  |
| *Paenibacillus sp. 10* | 3865 | 1 | 1 |  | MG871159 |
| *Serratia liquefaciens* | 3865 | 1 | 1 |  | MG871160 |
|  |  |  |  |  |  |
| ***Dendropsophus rhodopeplus*** | **560** | **2** | **2** | **2** |  |
| *Pseudomonas entomophila* | 560 | 1 | 1 | 1 | MG859621 |
| *Serratia marcescens* | 560 | 1 | 1 | 1 | MG859620 |
|  |  |  |  |  |  |
| ***Engystomops freibergi*** | **560** | **1** | **1** |  |  |
| *Paenibacillus sp. 18* | 560 | 1 | 1 |  | MG871164 |
|  |  |  |  |  |  |
| ***Gastrotheca antoniiochoai*** | **2915** | **2** | **2** |  |  |
| *Paenibacillus sp. 21* | 2915 | 1 | 1 |  | MG871166 |
| *Pseudomonas psychrophila* | 2915 | 1 | 1 |  | MG859642 |
|  |  |  |  |  |  |
| ***Gastrotheca excubitor*** | **3506** | **16** | **15** | **6** |  |
| *Acinetobacter radioresistens* | 3340 | 1 | 1 |  | KY742775 |
| *Aeromonas hydrophila* | 3695 | 1 | 1 |  | KY742769 |
| *Chryseobacterium sp. 6* | 3695 | 1 | 1 |  | KY742765 |
| *Hafnia alvei* | 3340 | 1 | 1 |  | KY742773 |
| *Janthinobacterium lividum* | 3695 | 1 | 1 | 1 | KY742771 |
| *Microbacterium sp.* | 3340 | 1 | 1 |  | KY742777 |
| *Pseudomonas constantinii* | 3340 | 1 |  |  | MG859613 |
| *Pseudomonas sp. 1* | 3695 | 1 | 1 | 1 | KY742768 |
| *Pseudomonas sp. 18* | 3340 | 1 | 1 | 1 | KY742774 |
| *Pseudomonas sp. 2* | 3340 | 2 | 2 | 1 | KY742776 |
| *Pseudomonas sp. 34* | 3340 | 1 | 1 | 1 | KY742779 |
| *Rahnella aquatilis* | 3695 | 1 | 1 | 1 | KY742767 |
| *Serratia sp. 1* | 3695 | 1 | 1 |  | KY742766 |
| *Serratia sp. 2* | 3695 | 1 | 1 |  | KY742770 |
| *Sphingomonas sp. 1* | 3340 | 1 | 1 |  | KY742778 |
|  |  |  |  |  |  |
| ***Gastrotheca nebulanastes*** | **2868** | **9** | **9** | **1** |  |
| *Bacillus simplex* | 2850 | 1 | 1 |  | KY742757 |
| *Bacillus sp. 2* | 2850 | 1 | 1 |  | KY742756 |
| *Microbacterium oxydans* | 2915 | 1 | 1 |  | KY742772 |
| *Paenibacillus sp. 2* | 2810 | 1 | 1 |  | KY742761 |
| *Pseudomonas sp. 25* | 2850 | 1 | 1 | 1 | KY742758 |
| *Pseudomonas sp. 4* | 2790 | 1 | 1 |  | KY742760 |
| *Pseudomonas sp. 5* | 2915 | 1 | 1 |  | KY742762 |
| *Pseudomonas sp. 6* | 2915 | 1 | 1 |  | KY742763 |
| *Pseudomonas sp. 7* | 2920 | 1 | 1 |  | KY742759 |
|  |  |  |  |  |  |
| ***Gastrotheca testudinea*** | **2100** | **2** | **2** |  |  |
| *Pseudomonas sp. 35* | 2100 | 1 | 1 |  | KY742781 |
| *Pseudomonas sp. 8* | 2100 | 1 | 1 |  | KY742780 |
|  |  |  |  |  |  |
| ***Hyalinobatrachium bergeri*** | **1411** | **7** | **7** |  |  |
| *Chryseobacterium indologenes* | 1030 | 1 | 1 |  | MG859645 |
| *Lysinibacillus boronitolerans* | 1030 | 1 | 1 |  | MG859643 |
| *Pseudomonas* | 1920 | 1 | 1 |  | MG859638 |
| *Pseudomonas denitrificans* | 1030 | 1 | 1 |  | MG859644 |
| *Pseudomonas sp. 24* | 1920 | 2 | 2 |  | MG859636-7 |
| *Pseudomonas sp. 26* | 1030 | 1 | 1 |  | MG859646 |
|  |  |  |  |  |  |
| ***Hypsiboas gladiator*** | **1414** | **14** | **11** | **5** |  |
| *Arthrobacter sp. 1* | 1410 | 1 | 1 |  | MG859560 |
| *Chryseobacterium* sp. | 1410 | 1 | 1 |  | MG859550 |
| *Chryseobacterium hagamense* | 1410 | 1 | 1 |  | MG859559 |
| *Chryseobacterium lactis strain KC1864* | 1410 | 2 | 2 |  | MG859557-8 |
| *Paenibacillus sp. 4* | 1450 | 1 |  |  | MG871152 |
| *Paenibacillus sp. 5* | 1450 | 1 | 1 |  | MG871152 |
| *Rahnella sp. 2* | 1410 | 2 | 1 | 1 | MG859555-6 |
| *Sphingobacterium faecium* | 1350 | 1 | 1 | 1 | KY742764 |
| *undetermined 1* | 1420 | 1 |  |  |  |
| *undetermined 2* | 1420 | 1 | 1 | 1 |  |
| *undetermined 3* | 1420 | 1 | 1 | 1 |  |
| *undetermined 4* | 1420 | 1 | 1 | 1 |  |
|  |  |  |  |  |  |
| ***Hypsiboas punctatus*** | **560** | **3** | **2** |  |  |
| *Chromobacterium sp.* | 560 | 1 |  |  | MG859617 |
| *Paenibacillus sp. 17* | 560 | 1 | 1 |  | MG859618 |
| *Pluralibacter pyrinus* | 560 | 1 | 1 |  | MG859616 |
|  |  |  |  |  |  |
| ***Adenomera andreae*** | **560** | **1** | **1** |  |  |
| *Paenibacillus sp. 16* | 560 | 1 | 1 |  | MG871163 |
|  |  |  |  |  |  |
| ***Noblella pygmaea*** | **2970** | **2** | **1** |  |  |
| *Paenibacillus sp. 25* | 2970 | 1 |  |  | N/A (low quality read) |
| *Pseudomonas sp. 11* | 2970 | 1 | 1 |  | N/A (low quality read) |
|  |  |  |  |  |  |
| ***Oreobates amarakaeri*** | **560** | **2** | **1** |  |  |
| *Enterobacteriaceae 2* | 560 | 1 | 1 |  | MG859624 |
| *Stenotrophomonas maltophilia strain SMB1* | 560 | 1 |  |  | MG859625 |
|  |  |  |  |  |  |
| ***Osteocephalus mimeticus*** | **1100** | **1** | **1** |  |  |
| *Bacillus circulans* | 1100 | 1 | 1 |  | MG871167 |
|  |  |  |  |  |  |
| ***Pristimantis cf. cruciocularis*** | **930** | **1** | **1** |  |  |
| *Pseudomonas monteilii* | 930 | 1 | 1 |  | MG859648 |
|  |  |  |  |  |  |
| ***Pristimantis cf. diadematus*** | **1065** | **1** | **1** | **1** |  |
| *Paenibacillus sp. 24* | 1065 | 1 | 1 | 1 | MG859649 |
|  |  |  |  |  |  |
| ***Pristimantis cf. platydactylus*** | **1938** | **10** | **8** |  |  |
| *Burkholderia sp. 2* | 1920 | 1 | 1 |  | MG859631 |
| *Chryseobacterium sp. 4* | 1960 | 1 | 1 |  | MG859574 |
| *Leifsonia shinshuensis* | 1920 | 1 | 1 |  | MG859632 |
| *Paenibacillus sp. 19* | 1920 | 1 | 1 |  | MG859630 |
| *Pseudomonas putida* | 1920 | 1 | 1 |  | MG859564 |
| *Stenotrophomonas maltophilia strain 24bKB2* | 1920 | 1 |  |  | MG859633 |
| *Stenotrophomonas sp. 2* | 1990 | 1 | 1 |  | MG859565 |
| *Stenotrophomonas sp. 3* | 1990 | 1 | 1 |  | MG859566 |
| *Stenotrophomonas sp. 5* | 1920 | 1 | 1 |  | MG859634 |
| *undetermined 1* | 1920 | 1 |  |  |  |
|  |  |  |  |  |  |
| ***Pristimantis danae*** | **1659** | **15** | **14** | **2** |  |
| *Acinetobacter sp. 3* | 1410 | 1 | 1 |  | MG859553 |
| *Agromyces aureus* | 1920 | 1 | 1 |  | MG859639 |
| *Chryseobacterium sp. 10* | 1410 | 1 | 1 |  | MG859548 |
| *Chryseobacterium sp. 11* | 1410 | 1 | 1 |  | MG859549 |
| *Cohnella sp.* | 1410 | 1 | 1 |  | MG859552 |
| *Erwinia sp.* | 1920 | 1 | 1 |  | MG859640 |
| *Herbaspirillum sp. 3* | 1920 | 1 | 1 |  | MG859641 |
| *Paenibacillus sp. 3* | 2000 | 1 | 1 | 1 | MG859571 |
| *Pantoea sp.* | 1450 | 1 |  |  | MG859581 |
| *Pseudomonas putida strain HD58* | 1920 | 1 | 1 |  | MG859569 |
| *Pseudomonas sp. 28* | 1410 | 1 | 1 |  | MG859551 |
| *Pseudomonas sp. 29* | 1410 | 1 | 1 |  | MG859554 |
| *Pseudomonas sp. 30* | 1920 | 1 | 1 |  | MG859570 |
| *Pseudomonas sp. 36* | 1920 | 1 | 1 | 1 | MG859568 |
| *Sphingobacterium sp. 2* | 1450 | 1 | 1 |  | MG859580 |
|  |  |  |  |  |  |
| ***Pristimantis lindae*** | **1920** | **1** | **1** |  |  |
| *Paenibacillus sp. 15* | 1920 | 1 | 1 |  | MG871162 |
|  |  |  |  |  |  |
| ***Pristimantis pharangobates*** | **2325** | **30** | **3** |  |  |
| *Acinetobacter sp. 1* | 2350 | 1 |  |  | MG859605 |
| *Aeromonas hydrophila strain ATCC 7966* | 2350 | 1 |  |  | MG859602 |
| *Bacillus sp. 1* | 1920 | 1 |  |  | MG859627 |
| *Chryseobacterium shigense* | 2790 | 1 |  |  | MG859536 |
| *Chryseobacterium sp. 3* | 1920 | 1 |  |  | MG859567 |
| *Chryseobacterium sp. 8* | 2350 | 1 |  |  | N/A (low quality read) |
| *Comamonas sp. 3* | 1920 | 1 |  |  | MG859629 |
| *Duganella sp.* | 1920 | 1 |  |  | MG859572 |
| *Enterobacteriaceae 1* | 2350 | 1 |  |  | MG859610 |
| *Exiguobacterium sp.* | 1920 | 1 |  |  | MG859628 |
| *Herbaspirillum sp. 2* | 1920 | 1 |  |  | MG859626 |
| *Janthinobacterium sp.* | 2790 | 1 | 1 |  | MG859532 |
| *Paenibacillus sp. 14* | 2790 | 1 |  |  | MG859533 |
| *Paenibacillus sp. 20* | 1920 | 1 |  |  | MG871165 |
| *Paenibacillus taichungensis strain LJ-23* | 2790 | 3 | 1 |  | MG859535-6 |
| *Pseudomonas reinekei* | 2350 | 1 |  |  | MG859600 |
| *Pseudomonas sp. 10* | 1960 | 1 |  |  | MG859573 |
| *Pseudomonas sp. 17* | 2350 | 1 |  |  | MG859604 |
| *Pseudomonas sp. 19* | 2350 | 1 |  |  | MG859609 |
| *Pseudomonas sp. 20* | 2350 | 1 |  |  | MG871165 |
| *Pseudomonas sp. 22* | 2790 | 1 | 1 |  | N/A (low quality read) |
| *Pseudomonas sp. 33* | 2350 | 1 |  |  | MG859612 |
| *Pseudomonas sp. 9* | 1920 | 1 |  |  | N/A (low quality read) |
| *Serratia myotis* | 2350 | 1 |  |  | MG859607 |
| *Serratia sp. 3* | 2350 | 1 |  |  | MG859601 |
| *Serratia sp. 4* | 2350 | 1 |  |  | MG859603 |
| *Sphingobacterium sp. 4* | 2350 | 1 |  |  | MG859606 |
| *undetermined 1* | 2350 | 1 |  |  |  |
|  |  |  |  |  |  |
| ***Pristimantis pluvialis*** | **930** | **2** | **2** |  |  |
| *Arthrobacter sp. 2* | 930 | 1 | 1 |  | N/A (low quality read) |
| *Pseudomonas sp. 27* | 930 | 1 | 1 |  | MG859647 |
|  |  |  |  |  |  |
| ***Pristimantis salaputium*** | **1398** | **19** | **14** | **1** |  |
| *Achromobacter sp.* | 1450 | 1 | 1 |  | MG859586 |
| *Averyella dalhousiensis* | 1400 | 2 | 1 |  | MG859582 |
| *Burkholderia sp. 1* | 1450 | 1 | 1 |  | N/A (low quality read) |
| *Burkholderia tropica* | 1450 | 1 | 1 |  | MG859585 |
| *Chryseobacterium sp. 1* | 1410 | 1 | 1 |  | MG859561 |
| *Comamonas jiangduensis* | 1350 | 1 | 1 |  | MG859590 |
| *Herbaspirillum sp. 1* | 1450 | 1 | 1 |  | MG859584 |
| *Lelliottia amnigena strain Lmb013* | 1350 | 1 | 1 |  | MG859589 |
| *Methylobacterium sp.* | 1450 | 1 |  |  | MG859587 |
| *Paenibacillus sp. 6* | 1450 | 1 |  |  | MG871154 |
| *Paenibacillus sp. 7* | 1350 | 1 |  |  | MG871155 |
| *Paenibacillus sp. 8* | 1350 | 1 | 1 |  | MG871157 |
| *Paenibacillus sp. 9* | 1350 | 1 |  |  | MG871158 |
| *Rahnella sp. 1* | 1350 | 1 | 1 |  | MG859588 |
| *Sphingobacterium sp. 3* | 1350 | 1 | 1 |  | MG871156 |
| *Sphingomonas sp. 2* | 1450 | 1 | 1 |  | MG859583 |
| *Stenotrophomonas sp. 1* | 1410 | 1 | 1 |  | MG859562 |
| *undetermined 1* | 1350 | 1 | 1 | 1 |  |
|  |  |  |  |  |  |
| ***Pristimantis sp.*** | **2350** | **1** | **1** |  |  |
| *Paenibacillus sp. 12* | 2350 | 1 | 1 |  | MG871161 |
|  |  |  |  |  |  |
| ***Pristimantis toftae*** | **1372** | **10** | **3** |  |  |
| *Chryseobacterium sp. 2* | 1410 | 1 | 1 |  | MG859563 |
| *Pseudomonas sp. 16* | 1450 | 1 |  |  | MG859579 |
| *Pseudomonas sp. 12* | 1350 | 1 |  |  | MG859591 |
| *Pseudomonas sp. 32* | 1350 | 1 | 1 |  | N/A (low quality read) |
| *Pseudomonas putida* strain JQ581 | 1350 | 1 |  |  | MG859592 |
| *Pseudomonas sp. 13* | 1350 | 1 |  |  | MG859593 |
| *Hafnia sp.* | 1350 | 1 |  |  | MG859594 |
| *Pseudomonas sp. 14* | 1350 | 1 |  |  | MG859595 |
| *Pseudomonas sp. 15* | 1350 | 1 |  |  | MG859596 |
| *Caulobacter sp.* | 1410 | 1 | 1 |  | MG859547 |
|  |  |  |  |  |  |
| ***Psychrophrynella usurpator*** | **2972** | **18** | **16** | **2** |  |
| *Chryseobacterium sp. 7* | 2975 | 1 | 1 |  | MG859526 |
| *Flavobacteriums sp. 1* | 2975 | 1 | 1 |  | MG859523 |
| *Mucilaginibacter sp.* | 2975 | 1 | 1 |  | MG859529 |
| *Paenibacillus sp. 1* | 2970 | 1 | 1 |  | MG859519 |
| *Paenibacillus sp. 11* | 2975 | 1 | 1 |  | MG859525 |
| *Paenibacillus sp. 13* | 2975 | 1 | 1 |  | MG859528 |
| *Paenibacillus sp. 2* | 2950 | 1 | 1 |  | MG859522 |
| *Pseudomonas azotoformans* | 2975 | 1 | 1 | 1 | MG859530 |
| *Pseudomonas poae* | 2975 | 1 | 1 |  | MG859538 |
| *Pseudomonas putida strain I3* | 2975 | 1 | 1 |  | MG859527 |
| *Pseudomonas sp. 21* | 2970 | 1 | 1 |  | MG859520 |
| *Pseudomonas sp. 23* | 2975 | 1 | 1 |  | MG859537 |
| *Pseudomonas tolaasii* | 2975 | 1 | 1 |  | MG859524 |
| *Sphingobacterium sp. 1* | 2970 | 1 | 1 |  | MG859521 |
| *undetermined 1* | 2973 | 1 |  |  |  |
| *undetermined 2* | 2973 | 1 |  |  |  |
| *undetermined 3* | 2973 | 1 | 1 |  |  |
| *undetermined 4* | 2973 | 1 | 1 | 1 |  |
|  |  |  |  |  |  |
| ***Rhinella manu*** | **2045** | **4** |  |  |  |
| *Burkholderia sp. 3* | 1920 | 1 |  |  | MG859635 |
| *Chryseobacterium vrystaatense* | 2080 | 1 |  |  | MG859614 |
| *Lelliottia amnigena strain JCM1237* | 2100 | 1 |  |  | N/A (low quality read) |
| *Stenotrophomonas sp. 4* | 2080 | 1 |  |  | MG859615 |
|  |  |  |  |  |  |
| ***Scinax ruber*** | **773** | **4** |  |  |  |
| *Comamonas testosteroni strain NBRC-14951* | 560 | 1 |  |  | MG859619 |
| *Comamonas testosteroni strain RD_MAAMIA_03* | 560 | 1 |  |  | MG859623 |
| *Salmonella enterica* | 560 | 1 |  |  | MG859622 |
| *Stenotrophomonas maltophilia strain GZUIFR-YC01* | 1410 | 1 |  |  | MG859546 |
|  |  |  |  |  |  |
| ***Telmatobius marmoratus*** | **3400** | **19** | **16** | **1** |  |
| *Acinetobacter sp. 2* | 3400 | 1 | 1 |  | MG859541 |
| *Aeromonas salmonicida* | 3400 | 1 | 1 |  | MG859540 |
| *Chryseobacterium sp. 5* | 3400 | 1 | 1 |  | MG859575 |
| *Chryseobacterium sp. 9* | 3400 | 1 | 1 |  | MG859544 |
| *Comamonas sp. 1* | 3400 | 1 | 1 |  | N/A (low quality read) |
| *Comamonas sp. 2* | 3400 | 1 | 1 |  | MG859576 |
| *Comamonas testosteroni strain T-X4A* | 3400 | 1 | 1 |  | MG859577 |
| *Flavobacteriums sp. 2* | 3400 | 1 | 1 |  | N/A (low quality read) |
| *Flavobacteriums sp. 3* | 3400 | 1 | 1 |  | MG859545 |
| *Hafnia alvei strain E2-10* | 3400 | 1 | 1 |  | MG859578 |
| *Herbaspirillum sp. 4* | 3400 | 1 | 1 |  | N/A (low quality read) |
| *Lactococcus piscium* | 3400 | 1 | 1 |  | MG859543 |
| *Paenibacillus sp. 22* | 3400 | 1 | 1 |  | N/A (low quality read) |
| *Paenibacillus sp. 23* | 3400 | 1 | 1 |  | MG859539 |
| *Pseudomonas fluorescens* | 3400 | 1 | 1 | 1 | MG859542 |
| *Pseudomonas sp. 31* | 3400 | 1 | 1 |  | N/A (low quality read) |
| *undetermined 1* | 3400 | 1 |  |  |  |
| *undetermined 2* | 3400 | 1 |  |  |  |
| *undetermined 3* | 3400 | 1 |  |  |  |
|  |  |  |  |  |  |
| **Total** | 2170 | 198 | 137 | 21 |  |
