# Supplementary material for: Widespread Elevational Occurrence of Antifungal Bacteria in Andean Amphibians Decimated by Disease: A Complex Role for Skin Symbionts in Defense Against Chytridiomycosis
Source: Front Microbiol. 2018 Mar 14;9:465. doi: 10.3389/fmicb.2018.00465 (PMC5861192; doi:10.3389/fmicb.2018.00465)
Supplement: Supplementary file 2 [file Table_2.DOCX]

**Table S2.** Prevalence of infection by the fungal pathogen *Batrachochytrium dendrobatidis* in amphibian hosts along the elevational gradient during the dry season of 2012.

| Elevation (m a.s.l.) | Prevalence | Lower | Upper |
| --- | --- | --- | --- |
| 550 | 0.20 | 0.05 | 0.36 |
| 950 | 0.64 | 0.44 | 0.84 |
| 1050 | 0.32 | 0.12 | 0.54 |
| 1150 | 0.45 | 0.25 | 0.66 |
| 1350 | 0.47 | 0.30 | 0.64 |
| 1450 | 0.38 | 0.27 | 0.49 |
| 1550 | 0.79 | 0.68 | 0.89 |
| 1950 | 0.56 | 0.47 | 0.65 |
| 2050 | 0.83 | 0.68 | 0.96 |
| 2150 | 0.86 | 0.67 | 1.00 |
| 2250 | 0.34 | 0.14 | 0.55 |
| 2350 | 0.54 | 0.40 | 0.68 |
| 2750 | 0.25 | 0.00 | 0.56 |
| 2850 | 0.06 | 0.00 | 0.23 |
| 2950 | 0.06 | 0.01 | 0.13 |
| 3350 | 0.47 | 0.29 | 0.64 |
| 3650 | 0.50 | 0.12 | 0.88 |
