# Supplementary material for: Widespread Elevational Occurrence of Antifungal Bacteria in Andean Amphibians Decimated by Disease: A Complex Role for Skin Symbionts in Defense Against Chytridiomycosis
Source: Front Microbiol. 2018 Mar 14;9:465. doi: 10.3389/fmicb.2018.00465 (PMC5861192; doi:10.3389/fmicb.2018.00465)

Figure S1. Ordination plot of phyla of amphibian skin bacteria OTUs using non-metric multidimensional scaling. Polygons join communities of hosts living at similar elevations (elevation classes of 500 m, see methods), coded by color from brown (540–999 m) to blue (3500–3865 m).

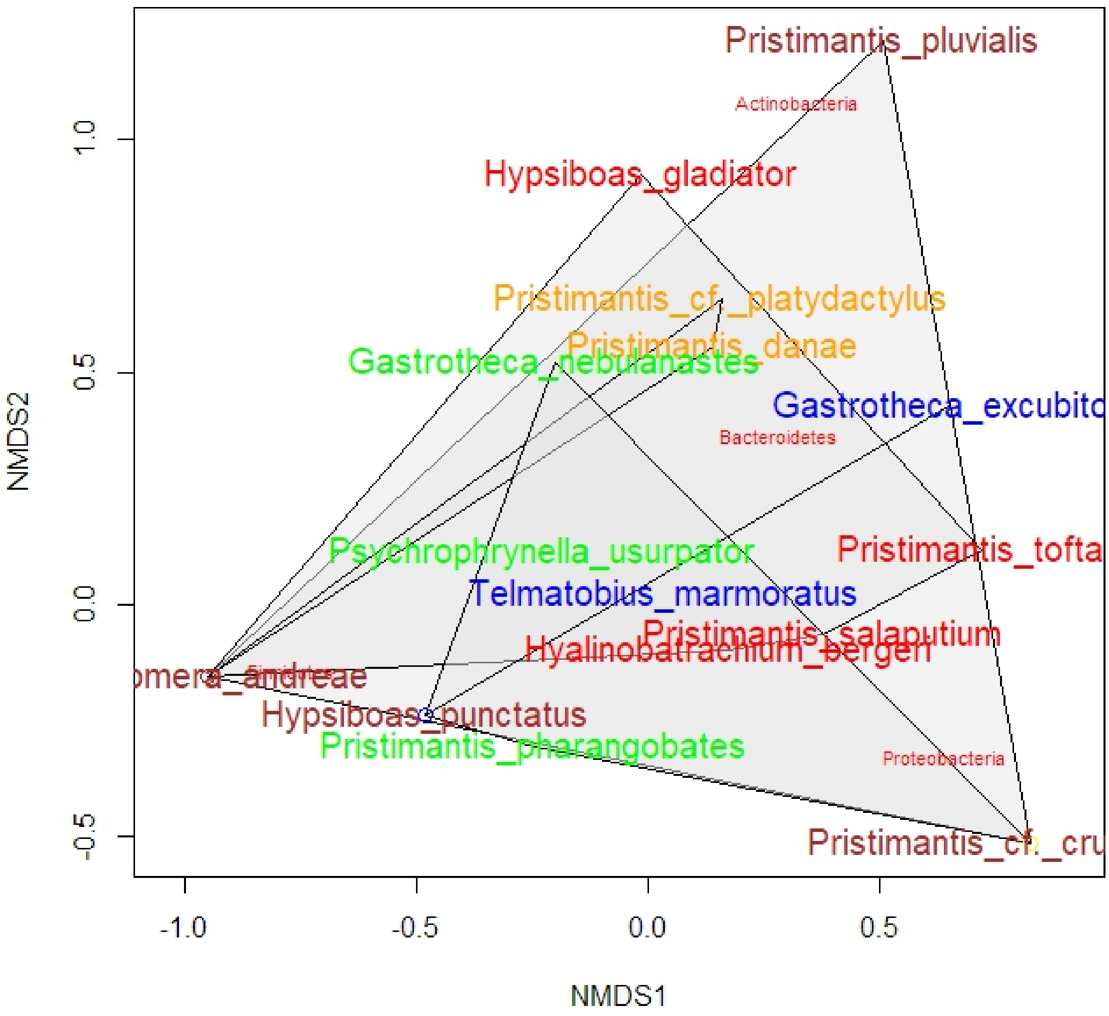

Supplement: Supplementary file 3 [file Image_1.PDF]
